# Performing a task with a friend does not change semantic processes but preparation: a social N400 and CNV event-related potential study

**DOI:** 10.3389/fpsyg.2025.1475106

**Published:** 2025-03-19

**Authors:** Sujata Sinha, Ashley Chau-Morris, Milena Kostova, J. Bruno Debruille

**Affiliations:** ^1^Department of Neurosciences, Faculty of Medicine, McGill University, Montréal, QC, Canada; ^2^Research Center of the Douglas Mental Health University Institute, Montréal, QC, Canada; ^3^Department of Psychiatry, Faculty of Medicine, McGill University, Montréal, QC, Canada; ^4^UR Paragraphe, Université Paris 8 Vincennes-Saint-Denis, Saint-Denis, France

**Keywords:** social N400s, semantic processing, participants with a friend, alones, common ground building, information sidelining

## Abstract

The N400 event-related potential (ERP) indexes the semantic processing of words. Recently, social N400 effects were reported: N400 amplitudes were found to be larger in the presence of a confederate. We tested whether this increase would be even larger in participants with friends (Pwfs). This was not the case: whether the words were coherent, incoherent or equivocal, N400s were not larger in Pwfs than in alones. According to the N400 inhibition hypothesis, the social N400 effects previously reported with confederates could then be due to the automatic sidelining of information that occurs when building a common ground with a stranger. Interestingly, contingent negative variations (CNVs) developed as the words had to be classified at the occurrence of an imperative stimulus that followed. PwFs had larger CNVs than alones, suggesting heightened preparation to this imperative stimulus. Unexpectedly, the larger this effect, the less confident PwFs were in their classifications. Given their higher levels of state anxiety before and after the experiment, it thus seems that the presence of someone else completing the same task, even if it is a friend, induces performance pressure, enhances anxiety and preparation, and diminishes self-confidence.

## 1 Introduction

The N400 is an event-related potential (ERP) that peaks around 400 ms after the onset of the presentation of meaningful stimuli, such as words, images, faces, and objects (Kutas and Hillyard, 1980; Nigam et al., 1992; Ganis et al., 1996; Hamm et al., 2002; Sitnikova et al., 2003; Kutas and Federmeier, 2011; Renoult et al., 2012; Schendan and Ganis, 2015; Draschkow et al., 2018). This is an electrically negative deflection that indexes semantic processes. Indeed, tasks focusing on non-semantic features, such as deciding whether a word is written with lower- or upper-case letters, elicit almost no N400 (Chwilla et al., 1995; Lien et al., 2021). This ERP is evoked by meaningful words, such as “apple.” Articles, such as “the,” elicit almost no N400. Moreover, little to no N400 is observed when the semantic processing that must be performed is minimal, e.g., when the occurrence of a word is fully primed by its context of occurrence. Conversely, large N400s are elicited by words that are less primed, such as “honey” in the sentence “He takes his coffee with cream and honey” (Kutas and Hillyard, 1980; Van Petten and Kutas, 1991; Weber-Fox and Neville, 2001; van Herten et al., 2006; Kutas and Federmeier, 2011).

The semantic processes underlying the N400 ERP have been proposed to be the activation or retrieval of semantic representations of stimuli that were not primed by preceding stimuli (Kiefer, 2002; see Kutas and Federmeier, 2011 for review). However, some findings suggest that this activation occurs earlier than N400. Compared with pseudowords that resemble actual words, such as “toble,” matched real words induce cortical activations before the N400 (Shtyrov et al., 2010; Macgregor et al., 2012). This is one of the reasons why some researchers view the N400 as an index of a later process, namely, of the integration of the memory representations activated by words within their context of occurrence (e.g., Hagoort et al., 2004; debates in Brouwer et al., 2017; Mantegna et al., 2019; Aurnhammer et al., 2021). On the other hand, recently, N400 processes have been proposed to correspond to adjustments required by predictive errors (e.g., Eddine et al., 2022; Rabovsky et al., 2018; Grisoni et al., 2021). The models derived from this view accurately predict N400 amplitudes in several situations.

Unexpectedly, social context also modulates the N400 amplitude. It was found to be larger in participants with a confederate than in those who were alone (Rueschemeyer et al., 2015; Westley et al., 2017; Jouravlev et al., 2019; Hinchcliffe et al., 2020; Forgács et al., 2022). In the first three studies, this N400 increase, called the “social N400,” was found (a) when participants knew they had privileged information, that is, extra information that was hidden from the confederate, and (b) when they also had to take the perspective of this confederate to decide whether or not the stimuli made sense. However, the social N400 was then also observed when participants did not have to take this perspective (Jouravlev et al., 2019; Forgács et al., 2022). Thus, at least in some settings, this perspective taking may occur automatically.

Interestingly, such social N400s were also found in conditions where both individuals received the same stimulus information (Hinchcliffe et al., 2020; Forgács et al., 2022). This may indicate that hiding privileged information from the confederate is not necessary for inducing social N400s. Perspective-taking may occur automatically in situations resembling everyday life activities, such as when two individuals witness the same event together (Sinha et al., 2023).

It is important to mention that in the above five studies (Rueschemeyer et al., 2015; Westley et al., 2017; Jouravlev et al., 2019; Hinchcliffe et al., 2020; and Forgács et al., 2022), social N400s were obtained when participants were with a stranger confederate, that is, with a person they did not previously know. If the social N400 actually indexes perspective taking, we hypothesized that this effect should be even larger in participants who are in the presence of someone they are familiar with, whose perspective they are already used to taking.

The present study first aimed at testing such an operational hypothesis by using the same experimental design as the one used by Sinha et al. (2023). It included three types of critical words: first, coherent words that can be predicted using their context of occurrence, second, words that are equivocal in their context and more difficult to predict. Third, incoherent words that were unpredictable. Participants were required to classify them accordingly and indicate the level of confidence they had in their response after each story. To prevent their motor potentials from contaminating ERPs in the N400 time window, they had to provide these two behavioral responses only once the imperative stimulus that followed each of these words occurred.

To prevent cognitive strategies that develop in one social context from contaminating strategies used in the other, a between-subject design was chosen. Approximately half of the participants were tested alone, while the remaining half were tested in the presence of a friend. This person was seated within the visual field of participants, as Sinha et al. (2023) reported no social N400 when the confederate is seated a bit behind participants and is therefore not in their visual field, suggesting that interaction, at least visual, is necessary for obtaining social N400s. We tested whether there was an increase in N400 amplitudes among participants with a friend compared to those completing the task alone.

Interestingly in Sinha et al. (2023), the imperative stimulus following each critical word always onsets 900 ms after the onset of the given word. Therefore, the experiment could induce contingent negative variations (CNVs; Walter et al., 1964; Tecce, 1972). Thus, it could also allow the detection of effects of social context on this ERP, which is a sensitive marker of expectancy (Cohen, 1969; Mento, 2017; Piedimonte et al., 2021), attention (Tecce, 1972; Brunia and van Boxtel, 2001), and preparatory processes (Brunia and van Boxtel, 2001; Schröder et al., 2024). It depends on cognitive effort and motivation to respond (Hamon and Seri, 1987). It serves as an index of proactive control, which involves sustaining goal-related information and fine-tuning attention, perception, and action systems to prepare before a cognitive task begins (Luo et al., 2024).

Like that of the N400, tha amplitude of the CNV was found to be influenced by the social context. For instance, Piedimonte et al. (2021) observed that participants with an observer exhibited smaller (i.e., less negative) CNVs than those who were alone. In contrast, Xu et al. (2020) found that socially excluded participants exhibit smaller CNVs in an AX-Continuous Performance Task (AX-CPT)^1^ than socially included participants.

In a third study (Zhao et al., 2024), in which an AX-CPT was also used, participants with high social anxiety were found to exhibit larger CNVs than those with low social anxiety.

These findings suggest that CNVs may depend on social contexts. In the present study, it might thus indicate whether the manipulation of the social context actually had an impact and allow us to determine whether this impact is consistent with the ones that were already found.

## 2 Materials and methods

### 2.1 Participants

The participants were recruited via advertisements on various social media websites, such as Facebook groups and marketplaces. Candidates filled out an eligibility questionnaire to see whether they could be selected to participate in the study. They had to be at least 18 years old, have normal or corrected-to-normal vision, and have completed college education. Candidates were excluded if they declared having a mental disorder at present or in the past or consuming psychotropic drugs more than once a week. Participants filled out the Edinburgh handedness inventory (Oldfield, 1971) and the French version of the national adult reading test (fNART; Mackinnon and Mulligan, 2005) to evaluate their verbal intelligence.

A total of 61 participants (39 women) were recruited to perform the task alone. They will be called “alones”. Of the 61, eight were rejected later due to poor EEG (see section 2.6 Data processing and measures). A total of 56 healthy participants (32 women) were selected to perform the experimental task with a close one, a friend, a sibling, or a partner. They will be designated here as the PwFs (participants with a friend). Of them, nine were later rejected due to poor EEG.

The PwFs and their friend had to know each other for at least 3 years. Their closeness was evaluated through a friendship questionnaire that was created for the experiment and that had to be filled out without communicating with the friend. This questionnaire consisted of 23 questions about the participant’s friend and 23 questions about the participant themselves. For example, the participant and their friend had to respond to “Where did your friend/sibling study?” and also “Where did you study?” Their answers were cross-verified, and each answer that matched was given a score of 1 (these questions, the participants’ and their friend’s answers, and the scores are available in Open Science Framework [OSF]). The overall friendship scores of the 47 PwFs ranged from 7 to 21 (mean = 14.6; standard deviation [SD] = 2.9). The sociodemographic data of these two groups are presented in Table 1.

**Table 1:** Sociodemographic data of the two participant groups.

Sociodemographic parameters	Alones	Participants with a friend (PwFs)
Mean ± SD of age (in years)	24 ± 3.9	22.3 ± 3.6
Sex (females/males)	39/14	32/15
Mean ± SD of the number of years of education	15.9 ± 1.8	15.3 ± 2.2
Mean ± SD of right-handedness (Edinburgh laterality)	80.3 ± 20.8	75.9 ± 18.9
Mean ± SD of verbal intelligence (fNART total score)	28 ± 4.4	26.9 ± 4.5
Mean ± SD state-anxiety score before the experiment (STAI Y-A)	28.2 ± 7.0	28.9 ± 8.5
Mean ± SD state-anxiety score after the experiment (STAI Y-A)	27.3 ± 6.7	27.7 ± 6.4

### 2.2 Consent

All participants read and signed an informed consent form that was accepted and approved by the Douglas Institute Research and Ethics Board (Douglas REB #12/12), which followed the guidelines of the Helsinki declaration.

### 2.3 Stimuli

As in Sinha et al. (2023), the stimuli for each participant included 180 short French stories featuring two characters interacting with each other (these stories are available in OSF). Each story consisted of two context-setting sentences and of a critical sentence. The last word of this critical sentence, called the target word, sets up the meaning of the story: coherent, incoherent, or equivocal. Coherent stories were logical, appropriate, and literal, whereas incoherent stories did not make sense. Importantly, the critical sentences were always coherent in themselves and non-ambiguous. The equivocal stories had an ambiguous meaning that was either ironic, humorous or deceptive (lies). For example, the story “Luc talks to his friend about his diet: I never eat smoked fish anyway. Because it is full of nicotine.” is equivocal, as the target word “nicotine” does not really explain why Luc would not eat smoked fish and just makes the story a humoristic one. Nevertheless, this humor is, for a large part, based on the surprise related to the contrast between this unexpected ending and the meanings of endings that could be predicted (e.g., bones, fat, or salt). Despite being disconfirmed by the actual ending, nicotine, these predictions might have to be kept by the participant for an accurate classification of the story.

Each story was created in the form of three conditions: coherent, incoherent, *and* equivocal (taken from Sinha et al., 2023). The mean cloze probabilities of the words ending the story were 0.53 (SD 0.35), 0.01 (SD 0.65), and 0.21 (SD 0.24) for the coherent, incoherent, and equivocal conditions, respectively. Some sentences were edited to suit the French spoken in the Quebec province of Canada. Cloze probabilities were not re-evaluated after the changes as the stimuli use was counterbalanced across participants.

The stimulus sequence for each participant consisted of 60 coherent, 60 incoherent, and 60 equivocal stories, as in Sinha et al. (2023). Each participant was presented with 60 × 3 = 180 stories. Each story appeared under only one of its three conditions. In other words, if a participant was presented with a story ending with a coherent meaning, the incoherent and equivocal forms of that story were not presented to him/her. These forms were presented to the other participants to counterbalance the stimulus material.

### 2.4 Procedure

**Figure 1:**
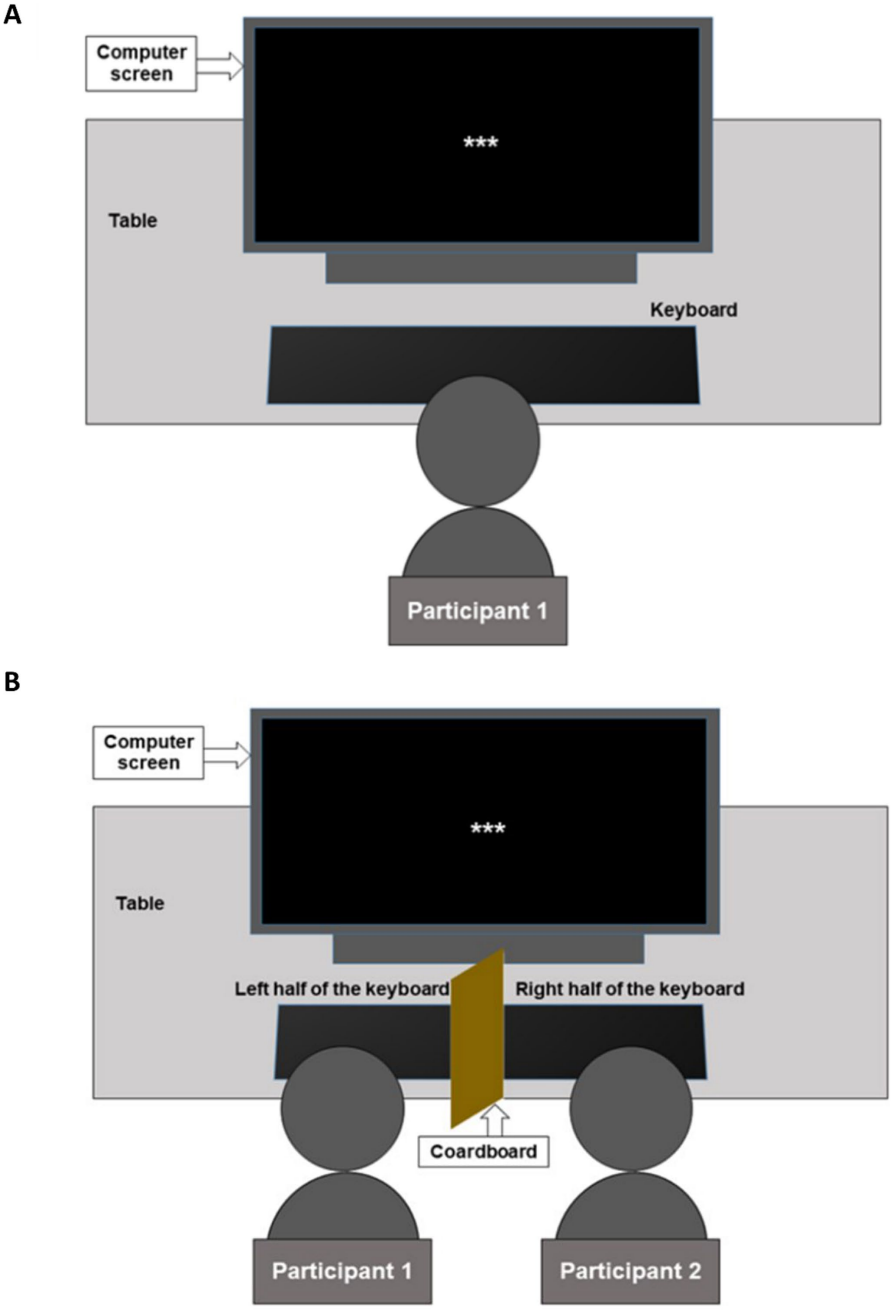
Experimental setup. **(A)** Alone participants were required to press the response keys on the right-hand side of the keyboard. **(B)** Participants with a friend (PwFs) were required to press response keys on the left side of the keyboard, while the friend responded by pressing keys on the right side of the keyboard, similar to the alones. The cardboard piece that divided the keyboard into two sides prevented participants from seeing the response given by their partner.

Upon arrival at the lab, the PwFs and their friend were asked to fill out the informed consent form and the friendship questionnaire separately, that is, without communicating, but in the presence of each other. The alones filled only the consent form. Given that an unfamiliar lab environment could induce more anxiety in the alones than in the PwFs, the initial state anxiety was controlled using the state part of the State–Trait Anxiety Inventory (the STAI Y-A; Spielberger, 1983). After EEG cap placement, the alones were seated alone in the experimental room for the task (see Figure 1A). The two PwFs were taken to the experimental room where they sat next to each other, facing the computer screen (see Figure 1B). While the EEG caps were placed, both were encouraged to talk and feel in the presence of each other. The entire experimental session consisted of a practice session, the real task, which included two breaks in the middle of it, and a debriefing session. The experimenter provided the same task instructions to the alone participants and to the participants with friends (PwFs). They were instructed not to move their head and body much, not to clench their jaw or contract face muscles during the experiment, and not to blink and make eye movements as much as possible when the words of the stories appeared on the screen.

They were then informed that they would be presented with stories on a computer screen. Each story consisted of three sentences: the first two context sentences appeared sequentially, and the final sentence was displayed word by word.

They were instructed to determine whether each story was coherent, incoherent, or equivocal based on the last sentence and to respond by pressing the keys labeled “1,” “2,” or “3” on the keyboard. Additionally, they were asked to indicate the level of confidence they had in their responses—by pressing one of the keys labeled from “1” to “5” on the keyboard, where “1” indicated the least confidence and “5” indicated the highest confidence.

The PwFs were asked to respond to the trials by pressing keys on the left side of the keyboard, while their friend had to press keys on the right side of the keyboard. To prevent seeing the responses of each other, a cardboard was used to divide the keyboard into two sides (see Figure 1B). The alones responded to the trials by pressing keys on the right side. At the end of the experiment, the PwFs and their friend filled out a second STAI Y-A and the debriefing questionnaire without communicating but in each other’s presence. The alones filled them out on their own.

**Figure 2:**
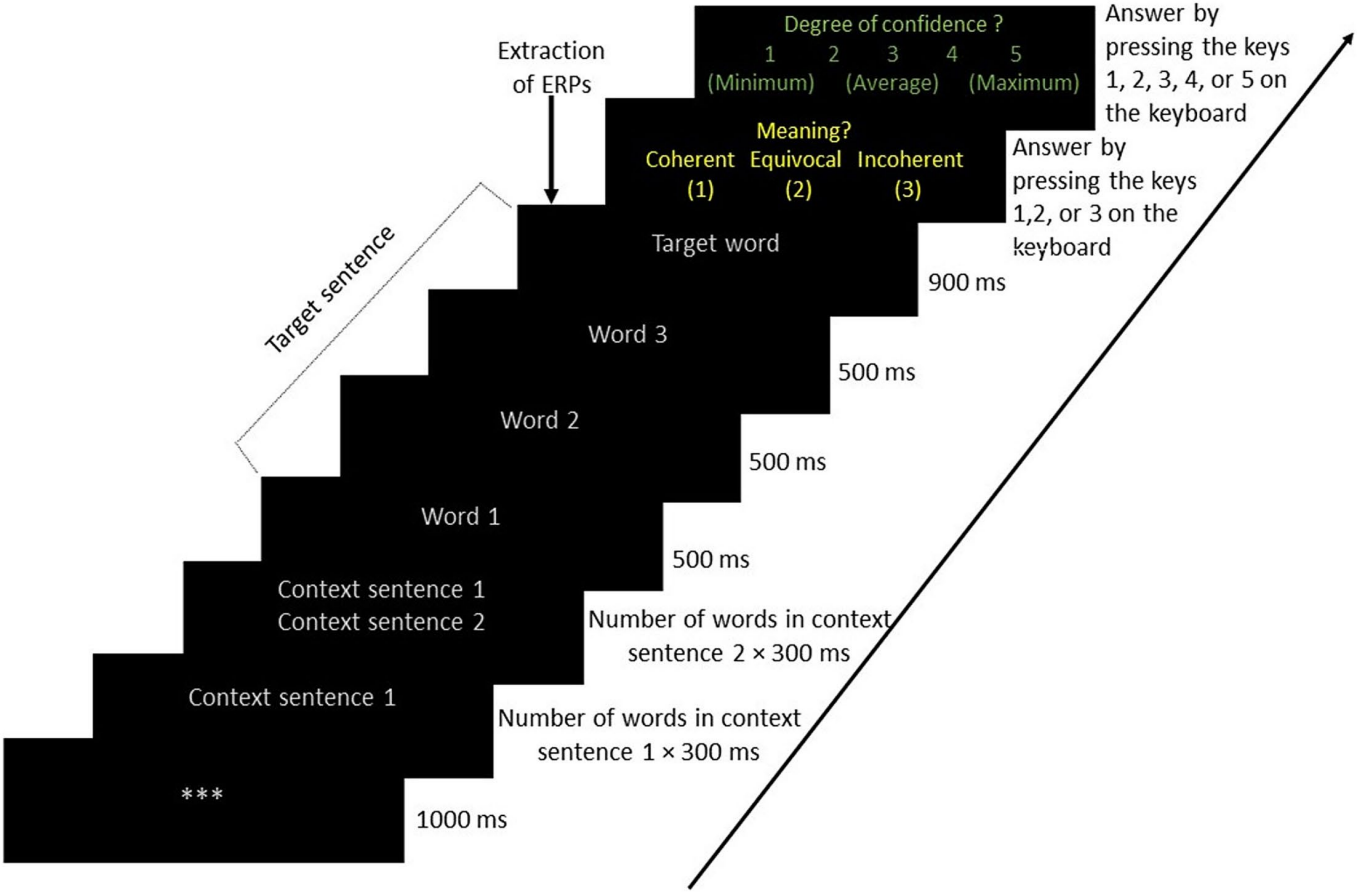
Sequence of stimuli presented to alones, participants with a friend, and friends with their timing. Stimuli appeared at the center of the computer screen.

The stimuli were presented to the alones and to the PwFs in a way that was similar to the one used in Sinha et al. (2023), as shown in Figure 2. Each trial started with 3 gray asterisks appearing at the center of the screen for 1,000 ms. They were followed by the first context sentence, written with 25-pt Arial font letters of a gray color on a black background. It appeared for a duration of 300 ms multiplied by the total number of words. Sentences including 5 words thus appeared for 1,500 ms, for instance. The second context sentence appeared with the same font and timing below the first context sentence. After these two context sentences, the critical sentence was presented word by word. Each of these words appeared for 500 ms except the last one, which appeared for 900 ms. Immediately following this, the question word: Meaning? (i.e., “Sens?” in French) appeared on the screen with three response options. These options were written in yellow (25-pt Arial font) on a black background below the question. The options were coherent, equivocal, and incoherent (“Cohérent,” “Équivoque,” and “Incohérent” in French, respectively). The participant then had to select one of them as quickly as possible by pressing the keys 1, 2, or 3 on the keyboard. The allocation of keys 1 and 3 to coherent and incoherent responses, respectively, was counterbalanced across participants. Key 2 was always used for the equivocal response. In the case of a friend pair, one participant (seated on the left side) responded using the alphanumeric keys while the other responded using the numeric keypad. Alones responded by pressing the numeric keypad keys.

Once participants had responded, and thus, in the case of PwFs, when both had responded, a second question occurred: Degree of confidence? (“Degré de confiance?” in French). This phrase was written in 25-pt green Arial font and appeared at the center of the screen with a black background. Below this question, a 5-point Likert scale (1 to 5) was displayed. The participant pressed one of the five keys to assess the level of confidence they had in the response they just provided. The friend who sat on the left side answered by pressing the Z, X, C, V, or B keys, where labels 1, 2, 3, 4, or 5 were stuck, respectively. The other friend on the right side and the alones did so by pressing the numerical keys 1, 2, 3, 4, or 5 on the right side.

### 2.5 Data acquisition

Behavioral responses were collected. EEGs were recorded from participants using a cap having 26 tin electrodes located according to the modified expanded 10/20 system (American Electroencephalographic Society, 1994). Their sites were F7/8, F3/4, Fc3/4, Ft7/8, C3/4, T3/4, Cp3/4, Tp7/8, P3/4, T5/6, O1/2, Fz, Fcz, Cz, and Pz. The right earlobe served as the reference, while the ground was taken from an electrode two centimeters in front of Fz. A 60-Hz electronic notch filter was used for both amplifier sets. The half-amplitude cut-offs of the high- and low-pass filters were set to 0.01 and 100 Hz, respectively. The EEG signals were amplified 10,000 times. They were then continuously digitized online at a sampling rate of 256 Hz and recorded in a single file with 26 × 2 = 52 electrodes for PwFs and a 26-channel file for alones.

### 2.6 Data processing and measures

These continuous EEGs were processed using the EEGLAB toolbox in Matlab 2019b (Delorme and Makeig, 2004). The PwF files were first split into one file for each of the two PwFs. Epochs of 1,200 ms duration starting 200 ms before the onset of each target word were extracted. They were baselined by computing their mean voltage in the −200 to 0 ms time window and by subtracting this mean from each of the points of the 1,200 ms epoch. Epochs with artifacts caused by eye movements and myograms were removed when voltages exceeded ±100 μV at F8/7 and ± 75 μV at any of the 24 other electrodes. Epochs that included amplifier saturation or analog-to-digital clippings were suppressed if one or more flat lines lasted beyond 100 ms.

Participants with less than 25 accepted trials in at least one of the 3 conditions (coherent, equivocal, or incoherent) were excluded. This left 53 alones and 47 PwFs. In alones, the mean numbers of accepted trials for the coherent, incoherent, and equivocal conditions were 40.4 (SD = 8.8), 41.8 (SD = 9.4), and 41.2 (SD = 9.2), respectively. For the PwFs, they were 42.7 (SD = 9.9), 43.5 (SD = 9.2), and 42.0 (SD = 9.9). The ERPs were computed by averaging the remaining EEG epochs for each of the 26 electrodes. Channels that appeared to lack ERPs were replaced by an average of the ERPs of the neighboring electrodes (see Supplementary Table S1).

The mean voltages of ERPs were measured from 300 to 500 ms post-target onset to study N400 amplitudes. This time window was selected based on a previous study using the same task (Sinha et al., 2023) and on previous N400 studies conducted with word stimuli (Holcomb, 1993; Kuperman et al., 1995; Berkum et al., 1999; Kutas and Federmeier, 2009; Lau et al., 2013; Kostova et al., 2014). For this time window, for each participant and for each of the three conditions, a centro-parietal region of interest (ROI) was chosen, as in Sinha et al. (2023). It included C3/4, Cz, Cp3/4, P3/4, and Pz electrodes. The ERPs of these electrodes were averaged.

Additionally, to test the unexpected differences observed between the 2 groups in the time window of the contingent negative variation (CNV), the mean ERP voltages were measured from 600 to 1,000 ms at all electrode sites. Seven participants with more than two standard deviations from the mean CNV measure at Fz were excluded as outliers.

### 2.7 Analyses

Omnibus mixed-model repeated-measures Analyses of Variances (ANOVAs) were conducted on response accuracies and confidence ratings. Each analysis had group (alones vs. PwFs) as a between-subject factor and condition (coherent vs. incoherent vs. equivocal) as a within-subject factor.

For N400s, a repeated-measure ANOVA was first run on the averages of the mean voltages of the ERPs of all ROI electrodes in the 300–500 ms time window. This test also had group as the between-subject factor and condition as the within-subject factor. The Greenhouse and Geisser (1959) procedure was used to compensate for variance heterogeneity across the three conditions. In this case, the original *F*-values and degrees of freedom are provided together with the corrected *p*-values. As the condition factor had a significant effect at each of the two time windows, Bonferroni-corrected (Weisstein, 2004) pairwise *post-hoc* comparisons were used to search for the source of the effect.

The results of the multivariate test of a repeated-measures ANOVA conducted on CNV measures were used to determine whether the unexpected CNV amplitude differences across the two groups of participants were significant. This analysis also used electrode and condition as within-subject factors. The Wilks’ lambda (Todorov and Filzmoser, 2010) values were also reported. The significant group × electrode interaction was decomposed to identify the sources of group differences, considering all electrodes.

Despite their larger CNVs frontocentral sites (F7, Fz, Cz, F4, and Fc4), PwFs, levels of confidence in their responses were significantly lower than those of the alones. Correlations were run to examine the relationship between these levels (averaged across the three conditions) and CNV measures (averaged across the specified sites) to see if these levels were related to the STAI-A questionnaire scores before and after the experiment.

## 3 Results

### 3.1 Behavioral data of alones and PwFs

The mean response accuracy (RA) of the 2 groups was 74.4% (SD = 11.09), and the mean confidence rating (CR) was 3.9 out of 5 (SD = 0.56). The scores for each of the three conditions are given in Table 2.

**Table 2:** Behavioral data of the two participant groups.

Conditions	Response accuracy percentage (RA) and confidence rating (CR, out of 5)	Alones (*n* = 53) Mean ± SD	Participants with a friend (PwFs, *n* = 47) Mean ± SD
Coherent	RA	79.9% ± 12.6%	78.3% ± 11.3%
	CR	4.2 ± 0.51	3.9 ± 0.49
Equivocal	RA	72.3% ± 13.5%	68.2% ± 16.2%
	CR	4.2 ± 0.35	3.8 ± 0.53
Incoherent	RA	74.3% ± 15.9%	73.2% ± 14.3%
	CR	4.0 ± 0.67	3.7 ± 0.37

The ANOVA performed to analyze RAs did not reveal any group effect or any interaction with condition (Supplementary Table S2A), that is, the two groups had similar RAs. However, the ANOVA run on CRs revealed that alones were more confident in their responses than PwFs (see Supplementary Table S2D). Condition had a significant effect on both RA and CR measures (*p* < 0.001; see Supplementary Tables S2C, D).

### 3.2 Electrophysiological data of alones and PwFs

**Figure 3:**
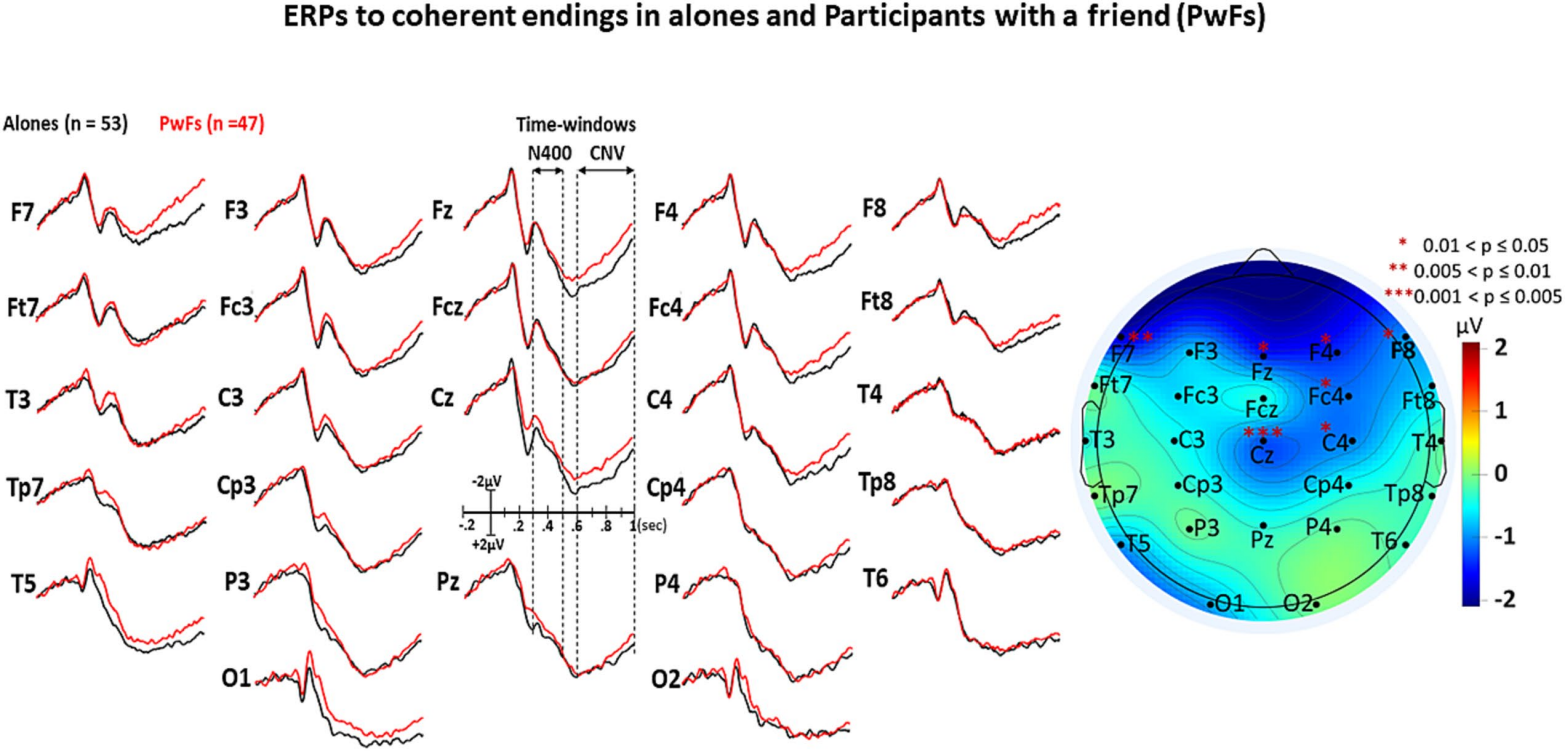
Grand average (GA) of the ERPs elicited by the target words (i.e., by the ending of each short story) of the *coherent* condition in the 2 groups, i.e., in the alones (black lines, *n* = 53) and in the participants with a friend, the PwFs (red lines, *n* = 47). The iso-voltage map displays the results of subtraction of the mean ERP voltages of PwFs from those of alones within the CNV time window, “staring” the electrodes where these results are significant.

**Figure 4:**
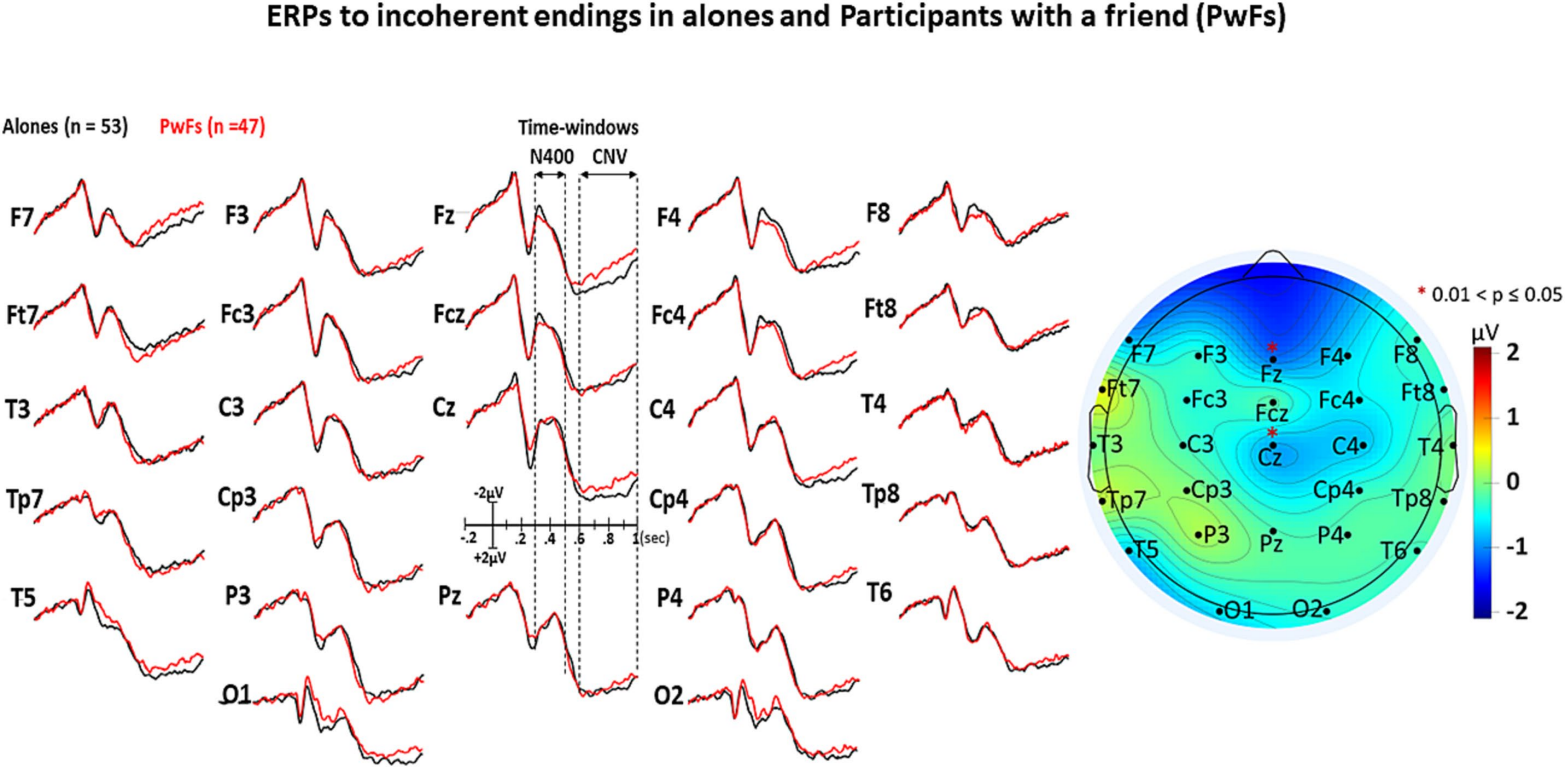
GAs under the incoherent condition. All other details are in Figure 3.

**Figure 5:**
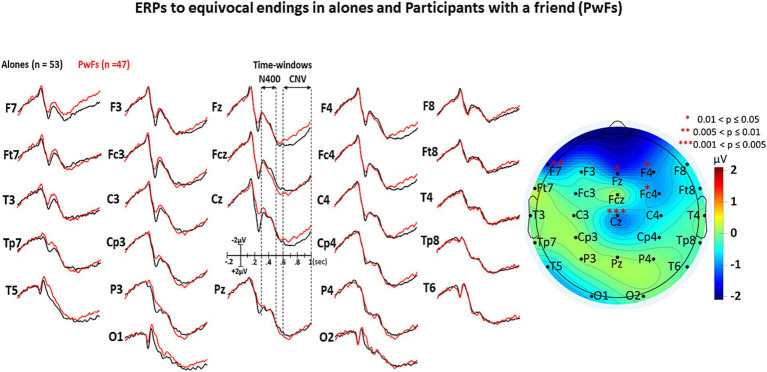
GAs under the equivocal condition. All other details are in Figure 3.

The visual inspection of Figures 3–5 shows that the grand averages of the ERPs of alones were similar to those of the PwFs in the N400 time window (i.e., from 300 to 500 ms). Unexpectedly, the CNVs appeared to be larger at some anterior and central electrodes in PwFs.

The ANOVA run with the N400 measures did not reveal any main effect of group or any interaction of this factor with condition (see Table 3A). In contrast, a main effect of condition was found, which was deconvolved by pairwise comparisons (see Table 3B). As expected, these comparisons showed significantly larger N400s for incoherent than (1) for equivocal and for coherent endings and then (2) for equivocal ones: these latter endings triggered N400s that were slightly larger than the coherent endings.

**Table 3A:** Results of repeated measures ANOVA run with the average of the mean voltages of the ERPs across ROI electrodes (C3/4, Cz, Cp5/6, Cp1/2, P7/8, P3/4, and Pz) in the N400 time window (300–500 ms) for alones and PwFs.

Factors Group (G: alones / PwFs) Condition (C: coherent / incoherent / equivocal)	df	F-values	*p*-values (Greenhouse–Geisser)	Effect size (η_p_^2^)	Observed power (for an alpha = 0.05)
G	1, 98	0.01	0.906	1.4 × 10^−**4**^	0.05
C	2, 196	77.53	2.080 × 10^−**24**^	0.4	1.00
G × C	2, 196	0.67	0.503	0.01	0.16

The analysis of CNV measures revealed that group interacted with electrode (see Table 4A). *Post-hoc* analyses were carried out to find the source of this interaction-The group effect (larger CNVs for PwFs than for alones) was found to be larger at frontocentral electrodes: F7, Fz, Cz, F4, and Fc4 (see Table 4B). There was also an interaction between condition and electrode (Table 4A).

**Table 3B:** Results of the post hoc pairwise comparisons decomposing the main effect of condition found in the N400 time window are reported in this table.

Condition pairs (Coh: coherent; Equi: equivocal; Incoh: incoherent)	Bonferroni-corrected *p*-values	Effect size (η_p_^2^)	Observed power (for an alpha = 0.05)
Coh vs. Equi	0.006	0.09	0.89
Coh vs. Incoh	2.722 × 10^−18^	0.55	1.00
Equi vs. Incoh	7.032 × 10^−14^	0.45	1.00

**Table 4A:** Results of the multivariate tests of the repeated-measures ANOVA run with the mean voltages of the ERPs of the CNV time window (600–1,000 ms) for alones and PwFs.

Factors Group (G: alones / PwFs) Condition (C: coherent / incoherent / equivocal) Electrode (E)	Wilks’ Lambda value	df	F-values	*p*-values (Greenhouse–Geisser)	Effect size (η_p_^2^)	Observed power (for an alpha = 0.05)
C	1.0	2, 90	1.61	0.205	0.04	0.33
E	0.2	25, 67	14.82	1.355 × 10^−18^	0.01	0.16
G × C	1.0	2, 90	0.24	0.790	0.8	1.00
G × E	0.6	25, 67	2.05	0.011	0.4	0.98
C × E	0.2	50, 42	3.36	5.132 × 10^−5^	0.8	1.00
G × C × E	0.5	50, 42	0.73	0.856	0.5	0.58

**Table 4B:** Decomposition of the G × E interaction of this table: electrodes at which the group effect was significant.

Electrode	df	F-values	*p*-values	Effect size (η_p_^2^)	Observed power (for an alpha = 0.05)
F7	1, 91	7.12	0.009	0.07	0.75
Fz	1, 91	6.33	0.014	0.07	0.70
Cz	1, 91	8.46	0.050	0.09	0.82
F4	1, 91	5.30	0.024	0.06	0.63
Fc4	1, 91	4.46	0.038	0.05	0.55

**Figure 6:**
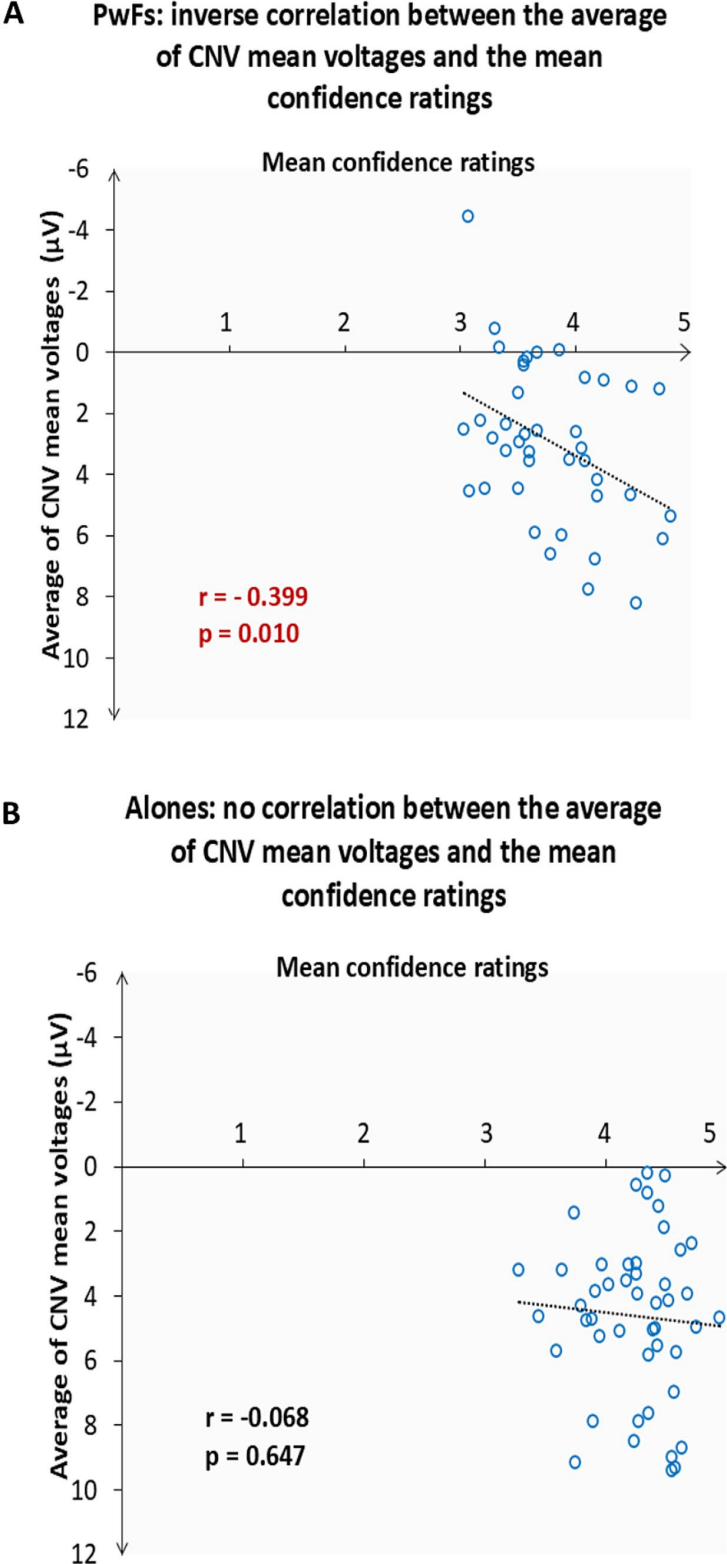
**(A)** Scatter plot showing the 41 of the 42 participants with a friend (PwFs) who were considered for the CNV analyses. One participant was excluded because the confidence score was more than two standard deviations away from the mean confidence rating of all conditions. The y-axis coordinate of each point is the average, for each participant, of their CNV mean voltage across the frontocentral ROI electrodes. The x-axis coordinate of each point is the mean level of confidence of the participant in their responses. The higher the ratings, the smaller (i.e., the less negative) the CNV mean voltages were. r is the Pearson’s correlation coefficient. The dotted line denotes the fit. **(B)** Scatter plot showing 47 of the 51 alone participants included in the CNV analyses. Four were outliers. No correlation was found for these alones.

The correlation test run to investigate the relationship between the average of the CNV mean voltages across the frontocentral electrode sites (F7, Fz, Cz, F4, and Fc4) and the mean confidence ratings of the three conditions revealed an inverse relationship (see Figure 6).

The second correlation revealed an inverse relationship between the state anxiety levels of the PwFs and their confidence ratings. This was observed both before (*r* = −0.337, *p* = 0.03) and after the experiment (*r* = −0.359, *p* = 0.02). In contrast, in alones, correlation was observed only after the experiment (*r* = −0.397, *p* = 0.006).

## 4 Discussion

In this study, we tested whether the increase of N400 amplitude (i.e., the social N400) that can be induced by the presence of a confederate seated in the visual field of participants would be even greater when this confederate was not a stranger but a person previously well-known to participants (a person referred to here as a friend). Such a result was not found. On the contrary, no social N400 effects were observed. The N400s of these participants (the PwFs, *n* = 47) did not significantly differ from those of participants who did the experiment alone (the alones, *n* = 53).

However, this negative finding fits the interpretation of the social N400s proposed by Sinha et al. (2023). This interpretation was derived from a particular view of N400 processes: the N400 inhibition view (Debruille, 1998, 2007; Debruille et al., 1996, 2008; Shang and Debruille, 2013; Sinha et al., 2023). According to this view, a part of the N400s index the inhibition of inappropriate representations that were activated by previous stimuli and by the early preconscious processing of the stimulus itself. For example, in the sentence “He takes his coffee with cream and honey,” the words prior to “honey” likely activated representations corresponding to “sugar.” These are inaccurate predictions that would be inhibited in order to integrate “honey” into the context of the sentence. Part of the N400s generated by unexpected words (such as “honey”) would be due to these inhibitions.

The Sinha et al.’s (2023) interpretation of social N400s was also based on the fact that the stimuli presented during an experiment can also activate representations of meanings, contexts, and situations that do not belong to this experiment, such as the context where the word has been perceived by that person before (e.g., Proust’s Madeleine; see Bray, 2013). Such private representations constitute privileged information that is known by the participant. Therefore, it may have to be inhibited to be set aside and to build a ground that is common to both the participant *and* the confederate. Indeed, such a sidelining has been demonstrated during this building (e.g., Samson et al., 2005; Brown-Schmidt, 2009; for a review, see Nokes-Malach et al., 2015).

This sidelining is not surprising. Social interactions require an understanding of the perspective of the individuals involved (see reviews, Schurz et al., 2013, 2020; Stietz et al., 2019; Samuel et al., 2023). This understanding focuses on shared information. Information held by only one of the persons involved in the interaction must be set aside in that person (Samson et al., 2005; Brown-Schmidt, 2009). Otherwise, there would be no common ground.

While these processes have been extensively studied during direct interactions between people, they could also occur when two people witness an event, such as in studies where social N400s were found (Rueschemeyer et al., 2015; Westley et al., 2017; Jouravlev et al., 2019). There, participants knew that the priviledged information they had was not held by the stranger confederate. The larger N400s in the presence of an unknown person may thus index the sidelining of this privileged information to build the common ground that tends to be automatically formed when in such a presence (Jouravlev et al., 2019).

The absence of social N400s in the present study suggests that the PwFs did not sideline the private-privileged information that was activated by words ending the stories. This sidelining would not have occurred because common grounds have already been established with their friend. The absence of social N400s between the two groups suggests a processing strategy that differs from the one that spontaneously occurs when meeting an unknown person. When with a friend, privileged information, such as private information would not be set aside but kept. For instance, because it can be shared with a friend and enrich the relationship.

This interpretation is strengthened by the fact that P1s and N1s were not smaller in PwFs than in alones. This allows us to eliminate the possibility that the absence of social N400s was caused by a lack of attention to PwFs. Indeed, had they been less attentive to stimuli than alones, they would have had such smaller P1s and N1s (e.g., Clark and Hillyard, 1996; Natale et al., 2006). These were not observed (see Figures 2–4).

Our protocol contrasted with three previous studies (Rueschemeyer et al., 2015; Westley et al., 2017; Jouravlev et al., 2019) and with experiments three to five of Forgács et al. (2022), in which privileged information was given to participants to induce a social N400 effect. However, it was like those of Hinchcliffe et al. (2020) and the first two experiments of Forgács et al. (2022), where no information disparity between participants and the confederate existed, and where social N400s were nevertheless found. Several other aspects of the N400 protocols used notably differ across these studies. Thus, the absence of social N400s observed here is unlikely to be due to the use of a different N400 protocol.

Neverthless, to determine with certainty that this absence was solely due to the placement of a friend within the participant’s peripheral view, it would be necessary to obtain social N400s with the same task in the presence of a stranger positioned within the participant’s visual periphery. Alternatively, another social N400 setup, such as Rueschemeyer et al. (2015), having a friend as the confederate could be used.

In contrast to the absence of social N400s, larger CNVs were observed in PwFs than in alones, indicating that the manipulation of the social context had an impact. These CNV differences were observed at frontocentral electrodes with a distribution that was consistent with that of the early CNV (Flores et al., 2009; Van Rijn et al., 2011; Herbst et al., 2015; Mento, 2017). This suggests that PwFs not only expected imperative stimuli but also mirrored the expectation of their friends, knowing that they too, would have to provide their behavioral responses immediately after the imperative stimulus occurred. Larger CNVs could also index the preparation of joint rather than isolated actions. Such joint actions require multiple participants to coordinate their actions with each other (Loehr et al., 2013; Painter et al., 2021; Bolt and Loehr, 2023).

These social context effects on the CNV are reminiscent of those reported by Xu et al. (2020), where larger CNVs were found in the socially included participants than in the excluded ones, which was interpreted as enhanced proactive control. Our results support the possibility that the presence of a friend increased the cognitive effort or heightened the expectations of the imperative stimulus and thus the preparation for its processing.

However, these results are in contrast with those of Piedimonte et al. (2021), in which participants with an observer exhibited smaller CNVs than participants who were alone. Obtaining effects in the reverse direction is probably due to the large differences existing between our and their tasks. In Piedimonte et al.’s (2021) task, the imperative stimulus was an aversive sequence of electrical shocks that had to be stopped. In other words, the task required the participants to shorten the imperative stimulus. In contrast, our task required participants to prepare for the correct response. There was no way to change the imperative stimulus.

Despite their larger CNVs, the confidence the PwFs had in their responses was significantly lower than that of the alones, whereas there was no difference in response accuracy. Moreover, a negative correlation was found between CNVs and these ratings: the smaller the CNVs were, the larger, that is the more negative the CNVs were. This correlation was observed only in the PwFs. Accordingly, it seems possible that the presence of a friend who was also completing the task concurrently induced pressure, thereby lowering confidence. Consistently, a negative correlation was found between state anxiety scores before and after the experiments and the confidence ratings. Performance anxiety might have increased preparedness for imperative stimuli, as revealed by their larger CNVs.

Surprisingly, participants’ state anxiety (measured before and after the experiment) had no significant effect on CNVs. This finding is in contrast to Zhao et al. (2024), who found that participants with high social anxiety exhibited larger CNVs than those with low social anxiety. In their study, social anxiety was assessed using the Chinese version of the Liebowitz Social Anxiety Scale, which specifically measures anxiety in social situations (He and Zhang, 2004). Therefore, the discrepancy between our findings and those of previous studies could be attributed not only to differences in experimental design but also to distinct anxiety measurement methods.

Whether social N400s could be linked to CNV effects remains a question, as CNVs were not examined in previous social N400 studies (Rueschemeyer et al., 2015; Westley et al., 2017; Jouravlev et al., 2019; Hinchcliffe et al., 2020; Forgács et al., 2022).

On the other hand, the current study did not specifically target age, sex, or gender, limiting the generalizability of the results. Future studies should focus on the potential effects of these factors and should also examine the effects of personality traits, such as schizotypy, which affect N400 amplitude. Second, the effects of the type of relationship (e.g., friends vs. work colleagues or members of the same team) could also be explored. Furthermore, one could test whether the same task would yield different results in a scenario in which participants and a friend or stranger perform the task cooperatively rather than individually.

## Data Availability

The datasets presented in this study can be found in online repositories. The names of the repository/repositories and accession number(s) can be found at: https://osf.io/kmxy2/?view_only=344dd85c54264f49b831a41c14cc1630.

## Footnotes

^1^The AX-Continuous Performance Task (AX-CPT) is a cognitive task that is often employed to evaluate aspects of cognitive control, particularly proactive and reactive control processes. In this task, participants are required to respond to a specific target sequence, usually a cue “A” followed by a probe “X,” while ignoring non-target sequences (e.g., “A” followed by non-X, “B” followed by X, or non-X; Braver et al., 2007). This task engages working memory and attentional control, requiring participants to maintain the contextual cue (“A”) in memory to respond accurately to the target (“X”; Braver et al., 2007).
