# Supplementary material for: Performing a task with a friend does not change semantic processes but preparation: a social N400 and CNV event-related potential study
Source: Front Psychol. 2025 Mar 19;16:1475106. doi: 10.3389/fpsyg.2025.1475106 (PMC11961880; doi:10.3389/fpsyg.2025.1475106)
Supplement: Supplementary file 1 [file Data_Sheet_1.pdf]

Supplementary Material

Performing a task with a friend does not change semantic processes but expectations:

A social N400 and CNV event-related potential study

Sujata Sinha[1,2], Ashley Chau-Morris[2, 3], Milena Kostova[4], J. Bruno Debruille[1, 2, 3, *]

*** Correspondence:** Corresponding Author: bruno.debruille@mcgill.ca

Supplementary Table S1. Channel recomputations by averaging the mean voltages of the neighboring electrodes

Table S1.A. Channel recomputations for alones (n=53)

Participant no	Channels recomputed	Formulae
1, 3, 5, 6, 8, 11, 14-25, 27, 28, 31, 33, 35-40, 42-44, 46, 48, 50-53	–	–
2	O1	O1 = O2
4	F7<br>F3	F7 = F8<br>F3=(Fc3+0.5F7+0.5Fz)/2
7	C4	C4=(Fc4+Cp4+0.5Cz +0.5T4)/3
9	C4	C4=(Fc4+Cp4+0.5Cz +0.5T4)/3
10	F7	F7 = F8
12	F4<br>F3	F4=(Fc4+.05Fz+0.5F8)/2<br>F3=(Fc3+0.5F7+0.5Fz)/2
13	F8	F8 = F7
26	Cz	Cz=(2Fcz+Pz)/3
29	Fc3	Fc3=(0.5Ft7 + F3+0.5Fcz+C3)/3
30	C3	C3=(Fc3+Cp3+0.5T3+0.5Cz)/3
32	Cz<br>P4<br>O1	Cz=(2Fcz+Pz)/3<br>P4= (C4 +0.5T5+0.5Pz)/2<br>O1=O2

34	Fcz	Fcz =(Fz+Cz+Fc3+Fc4)/4
41	C3	C3=(Fc3+Cp3+0.5T3+0.5Cz)/3
45	F7<br>F4<br>F3<br>Fz	F7=F8<br>F4=(Fc4+.05Fz+0.5F8)/2<br>F3=(Fc3+0.5F7+0.5Fz)/2<br>Fz=(F4+F3)/2
47	Fcz	Fcz =(Fz+Cz+Fc3+Fc4)/4
49	F7<br>F3<br>Fz<br>Fcz	F7=F8<br>F3=(0.5Fc3+0.5F7)/2<br>Fz=(F4+F3)/2<br>Fcz=(Fz+Cz+Fc3+Fc4)/4

Table S1.B. Channel recomputations for participants with a friend (n=47)

Participant no	Channels recomputed	Formulae
1, 3,6,7,11-14,16-19, 21-23, 26, 28, 37, 38, 42,44,45	–	–
2	C4<br>Cp4	C4=(Fc4+Cp4+0.5Cz +0.5T4)/3<br>Cp4 = (C4+P4)/2
4	C4<br>Cp4	C4=(Fc4+Cp4+0.5Cz +0.5T4)/3<br>Cp4 = (C4+P4)/2
5	F7<br>O2<br>T6<br>Tp8	F7=F8<br>O2=O1<br>T6=(T5 + O2)/2<br>Tp8=(T4+T6)/2
8	P3	P3=(Cp3+0.5T5+0.5Pz)/2
9	O1	O1=O2
10	P3	P3=(Cp3+0.5T5+0.5Pz)/2
15	Fc4	Fc4=(0.5Fcz+0.5Ft8+F4+C4)/3
20	P4<br>Fc4<br>C4	P4=(Cp4+0.5T6+0.5Pz)/2<br>Fc4=(0.5Fcz+0.5Ft8+F4+C4)/3<br>C4=(FC4+CP4+0.5Cz+0.5T4)/3

	F8<br>F7	
24	P4	P4=(Cp4+0.5T6+0.5Pz)/2
25	F8<br>F7<br>Fc4<br>Fc3	F8=(F4+Ft8)/2<br>F7= (F3+Ft7)/2<br>Fc4=(0.5Fcz+0.5Ft8+F4+C4)/3<br>Fc3=(0.5Fcz+0.5Ft7+F3+C3)/3
27	P3	P3=(Cp3+0.5T)+0.5Pz)/2
29	O1<br>F8	O1=O2<br>F8=F7
30	O1<br>F8	O1=O2<br>F8=F7
31	F8	F8=F7
32	T4	T4=(Ft8+Tp8)/2
33	F8<br>F7<br>Fz<br>Cz	F8=(F4+Ft8)/2<br>F7= (F3+Ft7)/2<br>Fz=(F3+F4)/2<br>Cz=(2Fcz+Pz)/3
34	Ft7<br>T6	Ft7=(F7+T3)/2<br>T6=(Tp8+O2)/2
35	Fc3	Fc3==(0.5Ft7+F3+0.5Fcz+C3)/3
36	Fc4<br>Fc3<br>F4<br>F3	Fc4=(0.5Fcz+0.5Ft8+C4)/2<br>Fc3=(0.5Fcz+0.5Ft7+C3)/2<br>F4=(Fc4+0.5Fz+0.5F8)/2<br>F3=(Fc3+0.5Fz+0.5F7)/2
39	Cz<br>P4<br>P3	Cz=(2Fcz+Pz)/3<br>P4=(Cp4+0.5T6+0.5Pz)/2<br>P3=(Cp3+0.5T5+0.5Pz)/2
40	Fz<br>O1	Fz=(F3+F4)/2<br>O1=O2
41	O1	O1=O2
43	Fz<br>Cz<br>F8<br>F7	Fz=(F3+F4)/2<br>Cz=(2Fcz+Pz)/3<br>F8=(F4+Ft8)/2<br>F7= (F3+Ft7)/2

46	Ft8<br>Fc3<br>Fc4<br>F4<br>F3	Ft8 = (F8+T4)/2<br>Fc3=(0.5Ft7+0.5Fcz+C3)/2<br>Fc4=(0.5Fcz+0.5Ft8+C4)/2<br>F4=(Fc4+0.5Fz+0.5F8)/2<br>F3=(Fc3+0.5F7+0.5Fz)/2
47	F8<br>T3<br>F4	F8=F7<br>T3 =(Ft7+Tp7)/2<br>F4=(Fc4+0.5Fz+0.5F8)/2

Supplementary Table S2. Results of the omnibus ANOVA for 2 groups (alones vs. PwFs) run with the mean response accuracy and the mean confidence ratings

Table S2.A. Results of the ANOVA on mean response accuracy

Number of tests (N)	Factors<br><br>Group (G, 2 levels)<br>Conditions (C, 3 levels)	df	F-values	p-values (Greenhouse-Geisser)	Effect size (η_p^2)	Observed Power (alpha=0.05)
3	G	1, 98	1.02	0.314	0.01	0.17
	C	2, 196	17.55	8.870×10^{-7}	0.15	0.99
	G × C	2, 198	0.61	0.513	0.01	0.14

Table S2.B. Results of the post-hoc pairwise comparisons decomposing the main effect of condition (C) in Table S2.A

Condition pairs (Coherent: Coh; Equivocal: Equi; Incoherent: Incoh)	Bonferroni-corrected p-value	Effect size (η_p^2)	Observed Power (alpha=0.05)
Coh vs. Equi	1.406×10^{-6}	0.23	1.00
Coh vs. Incoh	1.854×10^{-5}	0.20	1.00
Equi vs. Incoh	0.117	0.04	0.54

Table S2.C. Results of the ANOVA on mean confidence ratings

Number of tests (N)	Factors<br><br>Group (G, 2 levels)<br>Conditions (C, 3 levels)	df	F-values	p-values (Greenhouse-Geisser)	Effect size (η_p^2)	Observed Power (alpha=0.05)
3	G	1, 98	8.38	0.005	0.08	0.81
	C	2, 196	17.03	1.232×10^{-6}	0.20	0.99
	G × C	2, 196	1.30	0.272	0.01	0.26

Table S2.D. Results of the post-hoc pairwise comparisons decomposing the main effect of condition (C) in Table S4.C

Condition pairs (Coherent: Coh; Equivocal: Equi; Incoherent: Incoh)	Bonferroni-corrected p-value	Effect size (η_p^2)	Observed Power (alpha=0.05)
Coh vs. Equi	1.000	0.01	0.11
Coh vs. Incoh	3.424×10^{-5}	0.18	1.00
Equi vs. Incoh	5.746×10^{-5}	1.17	1.00
